# Moral Nutriment

**Published:** 1859-03

**Authors:** 


### MORAL NUTRIMENT.

Whose mind does not run far back into the past with sunny memories in reading the dear familiar lines—

> " In works of labor or of skill
> I would be busy, too,
> For Satan finds some mischief still
> For idle hands to do ?"

Lazy people eat more than the busy, at least for awhile, because it affords them enjoyment; it is a standing source of gratification, until they become dyspeptic, when every meal becomes more or less a torture.

But want of occupation has its attendant moral evils as well as physical. Idlers are nervous, fretful, peevish, cross. Ill-nature becomes a second nature, and they grumble, and complain, and whine from morning until night, with chance intervals of sunshine, but ever so transient.

One of the causes of the deep moral degradation of many sailors, is want of occupation in the interval of their " watches," especially in long voyages. We have many a time and oft been with them in the forecastle, from the full-rigged ship down through bark, and brig, and schooner, and tiny sloop, and have seen and heard all that was degrading in story and foul in act, profane and beastly, for want of occupation to lead them to higher things. The knowledge of this has led us for a long time past to preserve carefully all our religious exchanges, our agricultural papers, and the outside half-sheet of many weeklies, which, for safety in sentiment, purity of teaching, and courteousness of spirit, favorably compare with the religious press. For these a friend, whose heart is in the right place, comes regularly on the first of every month. No winter's frost or summer's fire by any chance keeps him away, although gray hairs are upon him, and his shadow is lengthening for the grave; and going down among the shipping, he hands them to the sailors of such vessels as are just weighing anchor, for the chance that some good sentiment may strike their attention in hours of quietude, and make them think of home, and sisters, and mother, and minister, the country church, the grave-yard close by, and of heaven; for even transient thoughts like these have a restraining, an elevating, purifying power. "These are the best things that come aboard for my men; they keep them out of mischief," said Captain ——, of the steamship Prince Albert, as the distributor jumped aboard and handed him a large bundle of reading matter. "We don't swear half so much when we have your papers to read," said a hardy jack tar. These two unvarnished statements are full of meaning; and we trust that our city readers will give them a practical turn by carefully preserving their religious papers, and other safe, transient, or loose reading matter, and send them free of charge to J. H., 283 Spring street, New York. A good religious newspaper ought not to be destroyed; nor, as we think, ought it to be laid away, to become moulded and worm-eaten, in the calculation of reading it again; for it is hiding in the napkin—it is hoarding up, instead of putting out at interest. We have many times copied a good article rather then mutilate the paper which contained it, thinking that if it did us good, it would be likely to do as great a good to some others, or a dozen others. Further, those who can write well for their favorite paper, who can throw off sentiments sparkling and pure, and short, terse, striking, and do not do it, are responsible to humanity and to God for the default. The making of a religious newspaper interesting, useful, influential, by reason of the sterling character of its reading matter, ought no more to be left to the editor, than the building up of an active, efficient church society, should be left wholly to the minister. Every man, woman, and child, ought to help him in all ways possible; and so ought the editor to have the sympathy, encouragement, and literary help of every reader who can thus contribute; for, next to the minister, a well-conducted religious newspaper is an instrument for present, extensive, enduring good, and they are essential to the times, as counteracting the malign influences which are scattered with a reckless hand by anonymous writers, who can stab from behind and in the dark, or by those who, leaving foreign countries for their country's good and their own safety, boldly solicit to be made the paid contributors of our best papers; and, having left home disappointed and depressed, take refuge in "liberal" views in doctrine and in drink, and pour out their infidelities and atheisms as largely as a sleepy public will allow; when at length, having lived up to their principles for a year or two, or more, their death and their *nom de plume*, with " real name," are for the first time made public; the " report" being—" Died" of maniæ potu, delirium tremen, drowned, run over by the cars at midnight, " died" by his own hand, by the visitation of God! Such are not a few of the men who, through the daily, the weekly, the monthly, and the quarterly, enter our parlors, and talk to our wives, and sons, and daughters, in gingerly infidelities—in gilded whoredoms. Men of a true humanity and a true progress! look to it that you write to counteract these poisons, and write as splendidly; look to it further, that your center tables be cleared of all this worse than trash, and assert and practice your right of a proper supervision of what your families are to read. There is " death in the pot," literary and moral, as in olden time there was in the culinary—moral death in many a fascinating novel and high-sounding magazine and " popular" weekly. Some reason was there in the declaration made to us lately by one of our sternest, most useful, and aged divines: " I allow no newspaper to be read in my family." Another, of a different profession, who was second to none in position and professional ability, since passed away with years and honors, said: " There is but one daily paper in New York that I consider fit to enter a family of daughters." Therefore, while one part of the community should watch the reading of their families with a jealous care, let those who can write well, pungently, and powerfully, feel it their duty to do what in them lies, to ensure that the literary *pabulum* of the people shall be unpoisoned—shall be prepared with materials that are morally pure, safe, and nutritious—that the reading for the masses be sound, truthful, and divine.

